# Preterm brain injury on term-equivalent age MRI in relation to perinatal factors and neurodevelopmental outcome at two years

**DOI:** 10.1371/journal.pone.0177128

**Published:** 2017-05-09

**Authors:** Margaretha J. Brouwer, Karina J. Kersbergen, Britt J. M. van Kooij, Manon J. N. L. Benders, Ingrid C. van Haastert, Corine Koopman-Esseboom, Jeffrey J. Neil, Linda S. de Vries, Hiroyuki Kidokoro, Terrie E. Inder, Floris Groenendaal

**Affiliations:** 1 Department of Perinatology, Wilhelmina Children’s Hospital and Brain Center Rudolf Magnus, University Medical Center Utrecht, Utrecht, The Netherlands; 2 Department of Neurology, Boston Children’s Hospital, Boston, Massachusetts, United States of America; 3 Department of Pediatrics, Nagoya University Graduate School of Medicine, Nagoya, Japan; 4 Brain & Mind Research Center, Nagoya University, Nagoya, Japan; 5 Department of Pediatric Newborn Medicine, Brigham and Women’s Hospital, Boston, Massachusetts, United States of America; Cincinnati Children's Hospital Medical Center, UNITED STATES

## Abstract

**Objectives:**

First, to apply a recently extended scoring system for preterm brain injury at term-equivalent age (TEA-)MRI in a regional extremely preterm cohort; second, to identify independent perinatal factors associated with this score; and third, to assess the prognostic value of this TEA-MRI score with respect to early neurodevelopmental outcome.

**Study design:**

239 extremely preterm infants (median gestational age [range] in weeks: 26.6 [24.3–27.9]), admitted to the Wilhelmina Children’s Hospital between 2006 and 2012 were included. Brain abnormalities in white matter, cortical and deep grey matter and cerebellum and brain growth were scored on T1- and T2-weighted TEA-MRI using the Kidokoro scoring system. Neurodevelopmental outcome was assessed at two years corrected age using the Bayley Scales of Infant and Toddler Development, third edition. The association between TEA-MRI and perinatal factors as well as neurodevelopmental outcome was evaluated using multivariable regression analysis.

**Results:**

The distribution of brain abnormalities and brain metrics in the Utrecht cohort differed from the original St. Louis cohort (*p* < .05). Mechanical ventilation >7 days (β [95% confidence interval, CI]: 1.3 [.5; 2.0]) and parenteral nutrition >21 days (2.2 [1.2; 3.2]) were independently associated with higher global brain abnormality scores (*p* < .001). Global brain abnormality scores were inversely associated with cognitive (β in composite scores [95% CI]: -.7 [-1.2; -.2], *p* = .004), fine motor (β in scaled scores [95% CI]: -.1 [-.3; -.0], *p* = .007) and gross motor outcome (β in scaled scores [95% CI]: -.2 [-.3; -.1], *p* < .001) at two years corrected age, although the explained variances were low (R^2^ ≤.219).

**Conclusion:**

Patterns of brain injury differed between cohorts. Prolonged mechanical ventilation and parenteral nutrition were identified as independent perinatal risk factors. The prognostic value of the TEA-MRI score was rather limited in this well-performing cohort.

## Introduction

With the increased use of term-equivalent age magnetic resonance imaging (TEA-MRI) as a biomarker for neurodevelopmental outcome among extremely preterm infants, there is a need for an accurate TEA-MRI scoring system to enable systematic and uniform evaluation of preterm brain abnormalities across cohorts.

Over the last decade, several TEA-MRI scoring systems have been developed to assess the degree of white matter (WM) and cortical grey matter (GM) injury[1,2] as well as to evaluate brain maturation.[3] In 2013, Kidokoro and colleagues extended a previous scoring system from their group to also incorporate evaluation of deep GM and cerebellar abnormalities.[1,4] In addition, several brain metrics were included to systematically account for impairments in brain growth. This extended scoring system provides a more comprehensive assessment of brain abnormalities on TEA-MRI, taking into account the increased awareness of the effect of deep GM and cerebellar injury on neurodevelopment.[5,6].

The global brain abnormality score (GBAS) by Kidokoro et al.[4] was recently shown to relate inversely to motor outcome as well as memory and learning performance at two and seven years of age, respectively; reported explained variances for outcome were, however, limited (<10%).[7,8].

The aims of this study were threefold. First, to apply the TEA-MRI scoring system by Kidokoro et al.[4] to a new cohort of extremely preterm infants from Utrecht; for this purpose, the distribution of brain abnormalities in the Utrecht cohort was compared with the St. Louis cohort, in whom the scoring system was evaluated initially.[4] Second, to identify perinatal factors independently associated with higher brain abnormality scores. Third, to evaluate the prognostic value of the TEA-MRI scoring system with respect to neurodevelopmental outcome at two years corrected age (CA).

## Methods

### Patients

Between October 2006 and December 2012, 332 extremely preterm infants (gestational age [GA] <28 weeks) were admitted to the level three Neonatal Intensive Care Unit of the Wilhelmina Children’s Hospital/University Medical Center Utrecht, The Netherlands and were eligible for participation in this prospective neuroimaging study approved by the institutional review board. Neonates with congenital anomalies (*n* = 5, 1.5%) were excluded. Fifty-two (15.7%) infants died before reaching TEA, and no parental informed consent was obtained for eighteen (5.4%) infants. Seven (2.1%) infants were examined on a 1.5 Tesla system, leaving 250 (75.3%) infants with a 3.0 Tesla TEA-MRI. We further excluded infants who were scanned at a postmenstrual age (PMA) ≥44 weeks (*n* = 2, 0.6%) or with severe motion artefacts on TEA-MRI (*n* = 1, 0.3%). Eight (2.4%) infants were lost to follow up at two years CA, leaving 239 (72.0%) infants with TEA-MRI and neurodevelopmental outcome data eligible for final inclusion.

Fifty-two (21.8%) of the 239 included infants were also included in a previous smaller study of 93 very preterm infants (gestational age [GA] <31 weeks), comparing the prognostic value of several neuroimaging modalities, including assessment of the TEA-MRI scoring system by Kidokoro et al.[4,7].

Permission from the medical ethical review committee of the University Medical Center Utrecht for the current study and oral informed parental consent for the MRI was obtained.

### Clinical variables

Maternal and neonatal charts were reviewed for demographic and perinatal characteristics. Socioeconomic status was based on maternal educational level.[9] Maternal educational level was classified as low, intermediate or high, depending on the highest educational grade.[10] Ethnicity was classified as Western, mixed, or non-Western, based on the ethnic background of both parents. Birth weight (BW) z-scores were computed according to the Dutch Perinatal registry reference data.[11] Postnatal events that were considered included days of mechanical ventilation, severe chronic lung disease (i.e. defined as the need for mechanical ventilation, positive airway pressure, and/or supplemental oxygen >30% at 36 weeks PMA[12]), inotropic support, patent ductus arteriosus requiring treatment with indomethacin or surgery, days of parental nutrition, perforated necrotizing enterocolitis, culture proven sepsis, germinal matrix-intraventricular hemorrhage (GMH-IVH; graded according to Papile et al.[13]), progressive post-hemorrhagic ventricular dilatation (PHVD; i.e. ventricular index >97th percentile according to Levene[14], anterior horn width >6mm or thalamo-occipital distance >24mm) requiring cerebrospinal fluid (CSF) drainage, and cystic periventricular leukomalacia (c-PVL; defined according to de Vries et al.[15]). GMH-IVH, PHVD, and c-PVL were diagnosed by sequential cranial ultrasound (cUS) examination, performed within six hours of admission, at least three times in the first week after birth, then weekly till discharge to a level two hospital, and again at TEA.

### TEA-MRI

MR images were acquired around TEA on a 3.0 Tesla MR system (Philips Healthcare, Best, The Netherlands) using a sense head coil. Infants were sedated with 50–60 mg/kg chloralhydrate by gastric tube. Until May 2008, conventional axial 3DT1-weighted imaging (repetition time [TR] = 9.4ms; echo time [TE] = 4.6ms; slice thickness = 2.0mm, no gap) and axial T2-weighted imaging (TR = 6293ms; TE = 120ms; slice thickness = 2.0mm, no gap) were performed. In June 2008, a new protocol was introduced, which involved coronal 3D T1-weighted imaging (TR = 9.5ms; TE = 4.6ms; slice thickness = 1.2mm, no gap) and coronal T2-weighted imaging (TR = 4847ms; TE = 150ms; slice thickness = 1.2mm, no gap).

#### Assessment of brain injury

WM, cortical and deep GM, and cerebellum were evaluated for the presence of brain abnormalities (MJNLB and LSV) and abnormal brain metrics (MJB and KJK) according to the scoring system by Kidokoro et al.[4] for T1- and T2-weighted TEA-MRI. Measurements were obtained using OsiriX (32-bit version, www.osirix-viewer.com), which allowed for free conversion to all planes. The GBAS was calculated as the sum of regional subscores and further classified as normal (0-≤3), mildly abnormal (4-≤7), moderately abnormal (8-≤11), and severely abnormal (≥12) according to Kidokoro et al.[4].

### Neurodevelopmental outcome

Neurodevelopmental outcome was assessed at either 24 or 30 months CA, depending on inclusion in a European study (www.i-med.ac.at/neobrain). Neurodevelopmental assessment was performed by a single developmental specialist (ICH), who was blinded for the TEA-MRI scores, using the cognitive, fine motor, and gross motor subtests of the Bayley Scales of Infant and Toddler Development, third edition (BSITD-III).[16] The composite and scaled scores corrected for premature birth were calculated (mean [standard deviation] in a normative population: 100 [15] and 10 [3], respectively). For motor outcome, only the scaled scores for gross and fine motor function were considered, as the composite motor score compromises both items and therefore provides less detail. The severity of cerebral palsy was graded according to the Gross Motor Function Classification System.[17].

### Statistical analysis

Data were analysed using IBM SPSS Statistics version 20 (SPSS Inc, Chicago, Illinois, USA). Measurements of the biparietal diameter, deep GM area, and transcerebellar diameter were corrected for PMA using linear regression analysis as described in the original paper (i.e. corrected measurement = original measurement+slope*[40-PMA]).[4] Corrected measures were used in subsequent analysis.

Perinatal characteristics and TEA-MRI scores of the Utrecht and St. Louis cohorts were compared using either a Chi-square or Fisher’s exact test for categorical variables and ANOVA for continuous variables.

The relationship between perinatal characteristics and TEA-MRI was explored using a Chi-square or Fisher’s exact test, logistic regression analysis, and multivariable regression analysis with TEA-MRI as dependent variable. This was done by hand in a forward manner with a *p*-value ≥.05 as exclusion criterion. All potential interactions were evaluated and statistically significant interactions between independent variables were added to the model.

The correlation between neonatal cUS and TEA-MRI was explored using multivariable regression analysis.

The association between TEA-MRI and neurodevelopmental outcome was evaluated using multivariable regression analysis. Given the number of missing data in the St Louis cohort, only the data from the Utrecht cohort were analysed. Results were adjusted for maternal education, non-Western ethnicity, female sex, GA, BW z-score, and test age (i.e. 24 or 30 months CA). A p-value < .05 was considered to be statistically significant; no corrections for multiple comparisons were needed using these models.

## Results

### Descriptive results

In total, 239 preterm neonates were included. Clinical characteristics and neurodevelopmental outcome data are presented in Table 1. Neurodevelopmental assessment was performed at a median (range) CA of either 24.0 (23.2–27.6) or 30.0 (29.5–30.9) months in 159 (66.5%) and 80 (33.5%) infants, respectively.

**Table 1:** Patient characteristics of the Utrecht and St. Louis cohorts.

	Utrecht cohort (*n* = 239)	St. Louis cohort (*n* = 97)	*P V*alue
**Neonatal characteristics^**a**^**			
Maternal age in years	31 (27–34; 21–42)	28 (23–33; 15–47)	.004
Maternal educational level^b^	Low: 75 (31.8)		
	Intermediate: 77 (32.6)		
	High: 84 (35.6)		
Ethnicity^b^	Western: 177 (74.1)		
	Mixed: 13 (5.4)		
	Non-Western: 49 (20.5)		
Gestational age in weeks	26.6 (25.9–27.4; 24.3–27.9)	27 (25–28; 23–32)	.39
Birth weight in gram	900 (760–1000; 455–1450)	930 (745–1120; 480–1600)	.03
Birth weight <2 SD	2 (.8)	6 (6.2)	.008
Female	177 (49.0)	54 (55.7)	.26
Multiple birth	75 (31.4)	33 (34.0)	.64
Antenatal corticosteroids (≥1 gift)	221 (93.6)^c^	80 (82.5)	.002
Caesarean section	110 (46.0)	68 (70.1)	< .001
Mechanical ventilation >7 days	120 (50.2)	30 (30.9)	.001
Oxygen at 36 weeks PMA	103 (43.1)	49 (50.5)	.22
Inotropics	102 (42.7)	33 (34.0)	.14
Persistent ductus arteriosus	108 (45.2)	39 (40.2)	.40
Parenteral nutrition >21 days	39 (16.8)^d^	38 (39.2)	< .001
Sepsis	96 (40.2)	29 (29.9)	.08
Perforated necrotizing enterocolitis	18 (7.5)	7 (7.2)	.92
**Sequential cranial ultrasound^**e**^**			
GMH-IVH	98 (41.0)		
Grade I	20 (8.4)		
Grade II	43 (18.0)		
Grade III	18 (7.5)		
PVHI	17 (7.1)		
PHVD requiring CSF drainage	20 (8.4)		
c-PVL	1 (0.4)		
**TEA-MRI^**a**^**			
Postmenstrual age in weeks	41.1 (40.7–41.6; 39.3–43.7)	38 (37–39; 36–42)	< .001
Weight in grams	3300 (2985–3630; 1685–4715)	2500 (2270–2745; 1490–3825)	< .001^f^
Head circumference in cm	35.2 (34.5–36.2; 30.0–39.0)	32.5 (31.9–33.5; 29.0–36.8)	< .001^g^
**Neurodevelopmental outcome at 24 and 30 months CA^**a**^**			
Cognitive composite score	105 (95–110; 60–145)	85 (70–100; 65–110)	< .001
Motor composite score	107 (100–112; 70–148)	85 (73–97; 58–107)	< .001
Fine motor scaled score	13 (11–14; 3–19)	8 (6–10; 2–15)	< .001
Gross motor scaled score	9 (8–10; 1–17)	7 (4–10; 1–11)	< .001
Cerebral palsy	6 (2.5)		

PVHI: periventricular hemorrhagic infarction; SD: standard deviation.

^a^ data are presented as either *n* (%) or median (interquartile range; range).

^b^ data were not comparible between both cohorts.

^c^ data were missing for three infants.

^d^ data were missing for seven infants.

^e^ sequential cUS data were not available for the St. Louis cohort.

^f^ adjusted for PMA in multivariable regression analysis: β (95% confidence interval, CI) for the St. Louis cohort: -395 gram (-600; -195; *p* < .001).

^g^ adjusted for PMA and weight at MRI-TEA in multivariable regression analysis: β (95% CI) for the St. Louis cohort: +.1 cm (-.5; .6; *p* = .85).

### Comparison with the original St. Louis cohort

**Fig 1:**
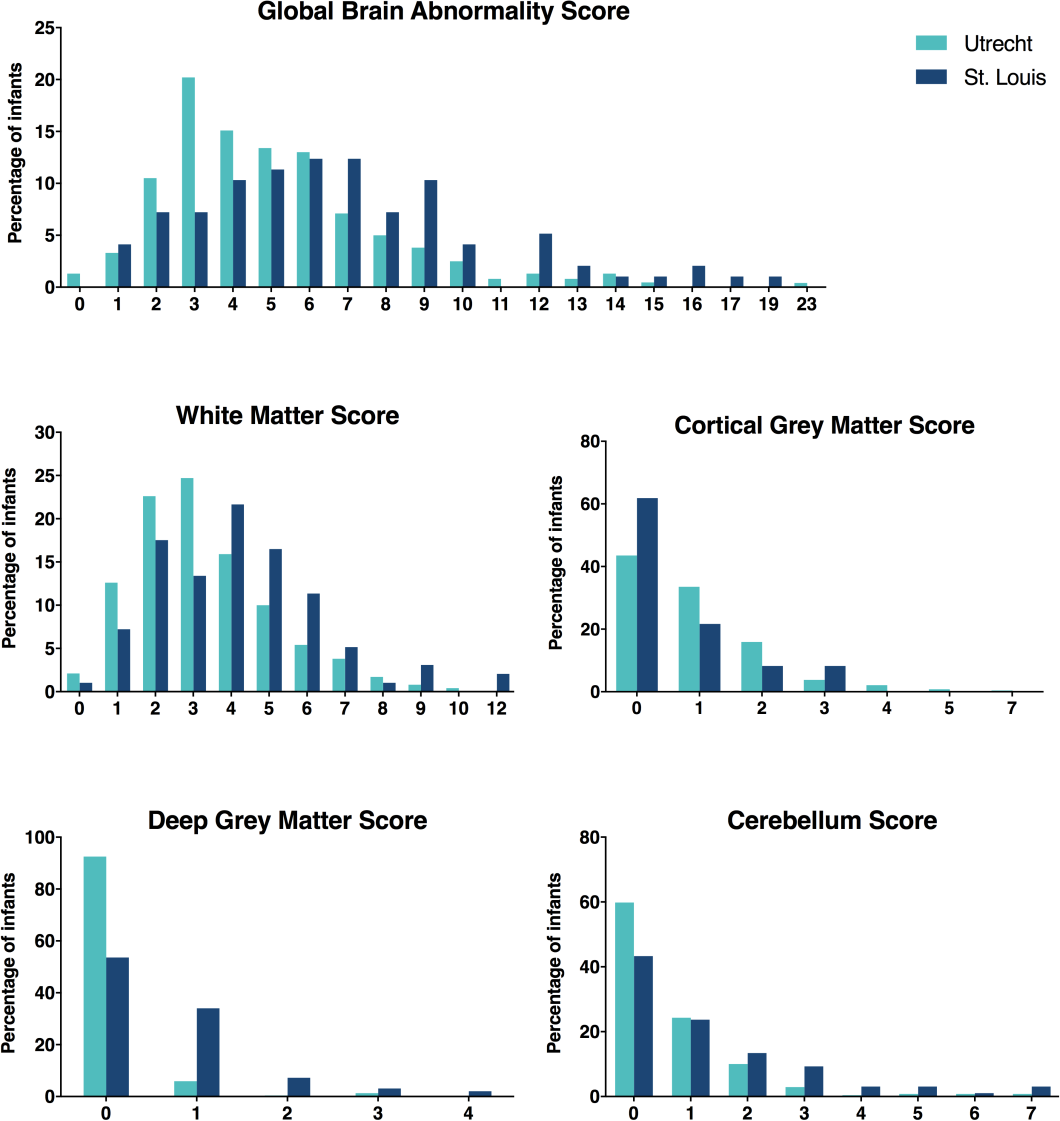
Distribution of the global brain abnormality score and regional subscores across the Utrecht (*n* = 239) and St. Louis (*n* = 97) cohorts. A left-sided shift of the curve can be appreciated for the Utrecht data.

A comparison of perinatal characteristics between the Utrecht cohort and St. Louis cohort (*n* = 97) is presented in Table 1. For more details regarding the latter cohort, we refer to the paper by Kidokoro et al.[4]Significant differences in the distribution of the GBAS and all regional subscores were observed between both cohorts (Fig 1; S1 Table). Overall, a smaller proportion of the Utrecht infants demonstrated moderate/severe brain injury (15.9% vs 35.0%; *p* < .001).

Substantial differences were also observed in the distribution of brain metrics (S2 Table). Compared to the Utrecht cohort, St. Louis infants demonstrated a relatively larger atrial size whereas measurements of the deep GM area and transcerebellar diameter, corrected for PMA at TEA-MRI, were reduced (*p* < .001). In both cohorts, deep GM and cerebellar measurements appeared to be interrelated. Deep GM area was 1.6 cm^2^ [95% confidence interval, CI: 1.4–1.8; *p* < .001] reduced in the St. Louis infants after adjustment for deep GM signal abnormalities, biparietal diameter, and transcerebellar diameter. Differences in cerebellar size between the Utrecht and St. Louis infants remained no longer statistically significant after correction for cerebellar signal abnormalities, biparietal diameter and deep GM area.

### Perinatal factors associated with TEA-MRI

In the Utrecht cohort, mechanical ventilation >7 days (β [95% CI]: 1.3 [.5; 2.0], *p* < .001) and parenteral nutrition >21 days (2.2 [1.2; 3.2], *p* < .001) were independently associated with a higher GBAS on TEA-MRI.

Infants with parenteral nutrition >21 days demonstrated a higher rate of cystic WM abnormalities (12.8% vs. 3.6%, *p* = .03), deep GM signal abnormalities (17.9% vs. 2.6%, *p* < .001), and cerebellar signal abnormalities (35.9% vs. 13.0%, *p* < .001).

Biparietal diameter, adjusted for body weight at TEA-MRI, was negatively related to both prolonged mechanical ventilation (-1.2 mm [-2.1; -.3], *p* = .008) and parenteral nutrition (-1.4 mm [-2.6; -.1], *p* = .03). Mechanical ventilation >7 days was also associated with a small reduction in deep GM area (-.4 cm^2^ [-.6; -.2], *p* < .001), adjusted for deep GM signal abnormalities, biparetial width, and transcerebellar diameter.

### Sequential cUS findings in relation to TEA-MRI

Grade II (1.5 [.7; 2.3]), grade III (2.5 [1.2; 3.8]), and grade IV (5.7 [4.3; 7.1]) GMH-IVH (*p* < .001) as well as PHVD requiring CSF drainage (2.0 [.7; 3.4], *p* = .007) were independently associated with a higher GBAS on TEA-MRI. GMH-IVH grade II-IV correlated with increased subscores of WM, deep GM, and cerebellum, whereas PHVD requiring CSF drainage was exclusively related to an increased cerebellum score.

### TEA-MRI and neurodevelopmental outcome

#### GBAS

The GBAS demonstrated an inverse relationship with cognitive, fine motor and gross motor performance at two years GA (Table 2, *p* < .01). Classification of the GBAS into four categories (i.e. normal, mildly, moderately, or severely abnormal) according to Kidokoro et al.[4] was only associated with gross motor outcome (β [95% CI] in scaled scores: -.6 [-1.0; -.3], *p* < .001, R^2^ = .152).

**Table 2:** Global brain abnormality score on TEA-MRI in relation to neurodevelopmental outcome in the Utrecht cohort (*n* = 239) according to multivariable regression analysis.

	β (95% CI)	*P* Value	R^2^
**Cognitive outcome**^**a**^**; composite score (mean [SD]: 100 [15]**^**d**^**)**			**.219**
(Constant)	104.1 (100.2; 108.0)		
Maternal education	3.0 (1.4; 4.7)	< .001	
Non-Western ethnicity	-8.3 (-11.7; -4.9)	< .001	
Female sex	3.6 (.8; 6.4)	.01	
Test age 30 months CA	-3.5 (-6.5; -.5)	.02	
Global brain abnormality score	-.7 (-1.2; -.2)	.004	
**Fine motor outcome**^**b**^**; scaled score (mean [SD]: 10 [3]**^**d**^**)**			**.130**
(Constant)	12.0 (10.9; 13.2)		
Maternal education	.6 (.3; 1.0)	.001	
Gestational age (centered at 24 weeks)	.4 (.1; .7)	.008	
Birth weight z-score	.4 (.1; .8)	.02	
Test age 30 months CA	-.9 (-1.5; -.2)	.01	
Global brain abnormality score	-.1 (-.3; -.0)	.007	
**Gross motor outcome**^**c**^**; scaled score (mean [SD]: 10 [3]**^**d**^**)**			**.178**
(Constant)	9.4 (8.7; 10.1)		
Maternal education	.6 (.2; .9)	.001	
Female sex	.6 (.0; 1.2)	.03	
Birth weight z-score	.5 (.2; .8)	.002	
Global brain abnormality score	-.2 (-.3; -.1)	< .001	

β: beta coefficient, representing the unit change in an outcome variable based on one unit change in the predictor variable; CI: confidence interval; SD: standard deviation.

^a^ the explained variance of maternal education, ethnicity, female sex, and test age (i.e. 30 versus 24 months CA) for cognitive outcome was .191 (*p* < .001); GA and BW z-score did not contribute to the model.

^b^ the explained variance of maternal education, GA, BW z-score, and test age for fine motor outcome was .102 (*p* < .001); ethnicity and female sex did not contribute to the model.

^c^ the explained variance of maternal education, female sex, and BW z-score for gross motor function was .108 (*p* < .001); ethnicity and GA did not contribute to the model.

^d^ i.e. in a normative population, according to the BSITD-III.

#### Subscores

Distinct associations were observed between the different subscores for WM, cortical and deep GM, and cerebellar injury and cognitive and motor outcome (i.e. in composite and scaled scores, respectively) at two years CA. Cognition was exclusively related to cerebellum scores (-1.8 [-2.9; -.6], *p* = .002, R^2^ = .223). Fine motor outcome was only associated with WM scores (-.2 [-.4; -.1], *p* = .004, R^2^ = .134). Gross motor outcome was independently related to WM (-.2 [-.4; -.1], *p* = .008) and deep GM (-1.2 [-1.9; -.5], *p* < .001) scores, with R^2^ = .206. We adjusted for the parameters mentioned in Table 2.

#### Brain metrics

**Fig 2:**
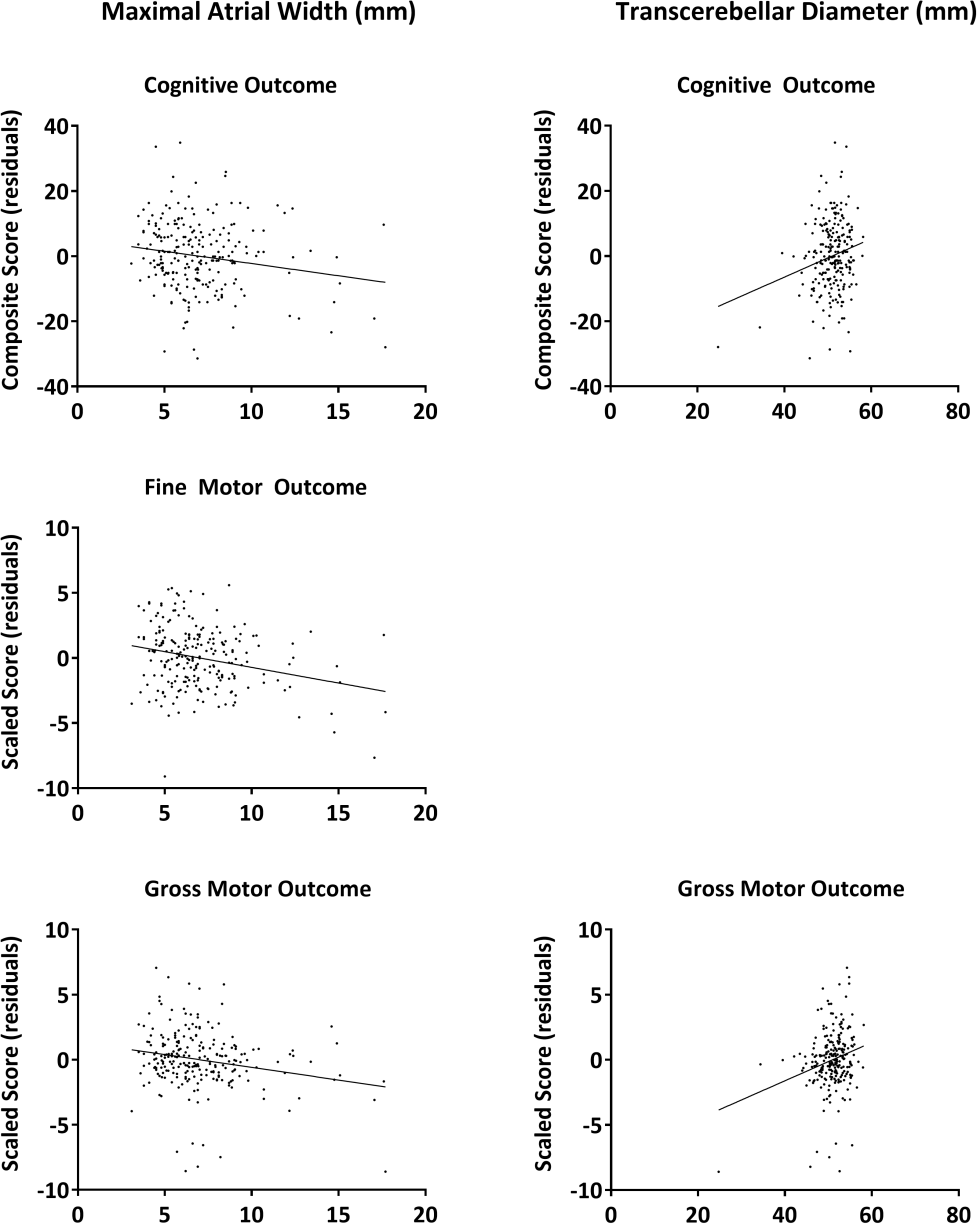
Association between brain metrics on TEA-MRI and neurodevelopmental outcome in the Utrecht cohort (*n* = 239). Presented are the residuals for cognitive outcome (i.e. corrected for maternal education, non-Western ethnicity, female sex, and test age), fine motor outcome (i.e. corrected for maternal education, GA, birth weight z-score, and test age), and gross motor outcome (i.e. corrected for maternal education, female sex, and birth weight z-score).

Independent associations with cognitive and motor outcome–in composite and scaled scores, respectively, and adjusted for the parameters mentioned in Table 2 –were exclusively observed for ventricular and cerebellar measurements on TEA-MRI (Fig 2). The maximal atrial width and transcerebellar diameter were independently associated with cognition (-.6/mm [-1.2; -.1] and .5/mm [.1; .9], respectively, *p* < .05; R^2^ = .239) as well as gross motor outcome (-.2/mm [-.3;-.0] and .1/mm [.0;.2], respectively, *p* < .05; R^2^ = .184). The maximal atrial width was also related to fine motor function (-.3/mm [-.4; -.1], *p* < .001; R^2^ = .165). After the exclusion of 102 (42.7%) infants with concomittant brain pathology on sequential cUS (i.e. GMH-IVH grade I-IV or c-PVL) or TEA-MRI (i.e. WM cysts or extensive signal abnormalities in WM, deep GM, or cerebellum), only the inverse association between the maximal atrial width and cognition remained statistically significant (-1.1/mm [-2.2; -.1], *p* = 0.03), with a trend for fine motor outcome (-.2/mm [-.5; .0], *p* = .06).

## Discussion

In this study, the recently developed scoring system for T1- and T2- weighted TEA-MRI by Kidokoro et al.[4] was evaluated in a large regional cohort of extremely preterm infants from Utrecht. The distribution of brain abnormalities and brain metrics was shown to differ from the original St. Louis cohort. In the Utrecht cohort, mechanical ventilation >7 days and parenteral nutrition >21 days were identified as risk factors for a higher GBAS on TEA-MRI in multivariable analysis. For the St. Louis cohort, these factors were also identified to relate to increased GBAS in univariate analysis.[4] TEA-MRI scores accounted for only a relatively small amount of variance in neurodevelopmental outcome at two years CA according to the BSITD-III.

Significant differences in the distribution of both the GBAS and regional subscores were observed between the Utrecht and St. Louis cohorts.[4] Overall, a smaller proportion of the Utrecht cohort demonstrated moderate/severe brain injury on TEA-MRI (15.9% vs 35.0%; Fig 1). There was a lower prevalence of ventricular enlargement in the Utrecht cohort. This may relate to differences in the incidence and severity of GMH-IVH and PHVD as well as periventricular WM injury with subsequent ex-vacuo dilatation. Since GMH-IVH, PHVD, and c-PVL were diagnosed differently across both cohorts (i.e. using sequential neonatal cUS in Utrecht vs. TEA-MRI in St. Louis), no definite conclusions can be drawn regarding the underlying pathophysiology.

Also striking was the relative reduction in deep GM and cerebellar size in the St. Louis cohort compared to the Utrecht cohort. This cannot be explained exclusively by the higher presence of focal signal abnormalities or by differences in brain size, hence suggesting different rates of regional brain growth impairments between cohorts. The prevalence of delayed gyral maturation was significantly lower in the St. Louis cohort (1.0% vs. 41.4%). This is likely due to the difference in median scan ages between the cohorts (38 vs. 41 weeks PMA) since the absence of tertiary folds in the inferior temporal and occipital lobes was the main reason to score infants as delayed in the Utrecht cohort, and this feature is not yet required at 38 weeks PMA.

As far as we could compare both cohorts, differences in the distribution of perinatal characteristics did not seem to be sufficient to explain the lower prevalence of brain injury in the Utrecht cohort. This suggests that other determinants, such as antenatal risk factors, socio-economic circumstances and access to antenatal care, might be involved. More intrinsic differences between populations, e.g. ethnicity and rate of teenage pregnancies, may also offer a partial explanation for the observed differences.

The negative effects of respiratory problems on preterm brain development, as observed in the present cohort, were previously acknowledged. Chronic lung disease has been shown to negatively impact brain volumes, cortical growth, and brain microstructure.[18–21] Prolonged mechanical ventilation and chronic lung disease have also been identified as independent risk factors for neurodevelopmental impairments, including cerebral palsy.[22–24].

Mechanisms underlying the observed adverse effects of prolonged parenteral nutrition are likely dual. On the one hand, parenteral nutrition itself can have a negative impact on brain growth and maturation.[25,26] On the other hand, preterm infants requiring prolonged parenteral nutrition tend to be the more severely ill infants in whom nutritional needs are often not optimally met, as evident in decreased growth rates of both body weight and head circumference.[27] Malnutrition following birth has previously been shown to delay microstructural development of the cortical GM.[25,28] The duration of parenteral nutrition has been reported to be negatively associated with regional brain volumes at TEA.[29].

In our extremely preterm cohort, the GBAS was inversely related to cognitive and motor outcome, although the amount of explained variance was small. Concerning the regional subscores, a negative association was observed between cognition and cerebellar abnormalities, which is in agreement with previous studies.[30–32] Fine motor outcome was related to WM injury, whereas gross motor outcome was associated with both WM and deep GM injury. WM abnormalities have previously been related to motor impairments.[33,34] Moderate/severe deep GM abnormalities in our cohort were invariably due to the presence of GMH-IVH grade III-IV and PHVD, which are known to be related to motor impairments.[35,36].

Regarding the brain metrics incorporated in the TEA-MRI score, associations with cognitive and motor performance were exclusively observed for ventricular and cerebellar measurements. The association between transcerebellar diameter and outcome was mainly determined by a few infants with a significantly reduction in cerebellar size due to a severe cerebellar haemorrage. Both ventriculomegaly, whether due to PHVD or ex-vacuo,[7,37] and cerebellar volume loss[30–32] were previously identified as risk factors for developmental impairments.

The strengths of the present study include the evaluation of the TEA-MRI scoring system in a large cohort of extremely preterm infants with high-quality neuroimaging and a follow-up rate of >97% of survivors. Some limitations should be mentioned. First, the limited prognostic value of the TEA-MRI scoring system observed in this study may relate to the overall relatively favourable outcome in the Utrecht cohort. In addition, evaluation of neurodevelopmental outcome at two years of age using the BSITD-III may not be sensitive enough to recognize more subtle neurodevelopmental deficits that first manifest at a later age.[38] This assumption is supported by the observed differences in cognitive and fine motor performance between infants evaluated at 24 versus 30 months of corrected age. Furthermore, TEA-MRI alone may not show the full extent of brain injury that occurred during the neonatal period.[7] Sequential early neuroimaging is necessary to fully appreciate the severity of GMH-IVH and subsequent PHVD as well as c-PVL.

Previous research also illustrated the limited capability of conventional TEA-MRI in particularly predicting cognitive outcome.[33,39–41] Additional MRI techniques such as diffusion tensor imaging, (resting-state) functional MRI or MR-spectroscopy, may provide more insight into the relationship between more subtle or diffuse patterns of brain injury and the spectrum of long-term neurodevelopmental deficits associated with preterm birth.

In conclusion, the scoring system by Kidokoro et al.[4] allows for a structured and comprehensive evaluation of brain abnormalities in preterm born infants at TEA. The need for prolonged mechanical ventilation and parenteral nutrition was found to be associated with a higher rate of brain abnormalities on TEA-MRI. The prognostic value of the TEA-MRI scoring tool in this relatively well-performing cohort appeared to be rather limited. Since patterns of brain abnormalities and impaired brain growth were shown to differ among preterm cohorts, we recommend further evaluation of the TEA-MRI scoring system and incorporated items in relation to long-term neurodevelopmental outcome across other preterm populations.

## Supporting information

S1 Dataset Original dataset used in this manuscript. (XLSX)

S1 Table Distribution of the global brain abnormality score and regional subscores on TEA-MRI across the Utrecht and St. Louis cohorts. (DOCX)

S2 Table Distribution of brain metrics incorporated in the TEA-MRI scoring system across the Utrecht and St. Louis cohorts. (DOCX)
