# Supplementary material for: Preterm brain injury on term-equivalent age MRI in relation to perinatal factors and neurodevelopmental outcome at two years
Source: PLoS One. 2017 May 9;12(5):e0177128. doi: 10.1371/journal.pone.0177128 (PMC5423624; doi:10.1371/journal.pone.0177128)
Supplement: S1 Table — (DOCX) [file pone.0177128.s002.docx]

| **S1 Table.** Distribution of the global brain abnormality score and regional subscores on TEA-MRI across the Utrecht and St. Louis cohorts. | | | |
| --- | --- | --- | --- |
|  | **Utrecht cohort**  **(*n*=239)** | **St. Louis cohort**  **(*n*=97)** | ***P* Value** |
| **Global brain abnormality score; median (range; IQR)** | **4 (0-23; 3-6)** | **6 (1-19; 4-9)** | **<.001** |
| **Global brain classification; *n* (%)** |  |  | **<**.**001** |
| Normal (0-3) | 85 (35.6) | 18 (18.6) |  |
| Mild (4-7) | 116 (48.5) | 45 (46.4) |  |
| Moderate (8-11) | 29 (12.1) | 21 (21.6) |  |
| Severe (≥12) | 9 (3.8) | 13 (13.4) |  |
|  |  |  |  |
| **WM score; median (range; IQR)** | **3 (0-10; 2-4)** | **4 (0-12; 2-5)** | **<.001** |
| **WM classification; *n* (%)** |  |  | **.01** |
| Normal (0-2) | 89 (37.2) | 25 (25.8) |  |
| Mild (3-4) | 97 (40.6) | 34 (35.1) |  |
| Moderate (5-6) | 37 (15.5) | 27 (27.8) |  |
| Severe (≥7) | 16 (6.7) | 11 (11.3) |  |
| ***Cystic lesions; n (%)*** |  |  | **.008** |
| None | 224 (93.7) | 90 (92.8) |  |
| Focal unilateral | - | 3 (3.1) |  |
| Focal bilateral | - | 1 (1.0) |  |
| Extensive unilateral | 14 (5.9) | 2 (2.1) |  |
| Extensive bilateral | 1 (.4) | 1 (1.0) |  |
| ***Focal signal abnormalities; n (%)*** |  |  | **.05** |
| None | 176 (73.6) | 77 (79.4) |  |
| Focal punctate | 56 (23.4) | 13 (13.4) |  |
| Extensive punctate | 5 (2.1) | 5 (5.2) |  |
| Linear | 2 (.8) | 2 (2.1) |  |
| ***Myelinisation; n (%)*** |  |  | **.005** |
| Posterior limb internal capsule + corona radiata | 160 (66.9) | 65 (67.0) |  |
| Only posterior limb internal capsule | 78 (32.6) | 26 (26.8) |  |
| Minimal-no posterior limb internal capsule | 1 (.4) | 6 (6.2) |  |
| ***Thinning corpus callosum; n (%)*** |  |  | **.02** |
| None | 86 (36.0) | 40 (41.2) |  |
| Partial^a^ | 152 (63.6) | 53 (54.6) |  |
| Global^a^ | 1 (0.4) | 4 (4.1) |  |
| ***Dilated lateral ventricles; n (%)*** |  |  | **<.001** |
| Both sides <7.5 mm | 158 (66.1) | 26 (26.8) |  |
| One side 7.5-<10 mm | 49 (20.5) | 19 (19.6) |  |
| Both sides 7.5-<10 mm / One side ≥10 mm | 24 (10.0) | 42 (43.3) |  |
| Both sides ≥10 mm | 8 (3.3) | 10 (10.3) |  |
| ***Volume reduction; n (%)*** |  |  | **.11** |
| Biparietal diameter^b^ ≥77 mm | 35 (14.6) | 21 (21.7) |  |
| Biparietal diameter^b^ 72-<77 mm | 107 (44.8) | 30 (30.9) |  |
| Biparietal diameter^b^ 67-<72 mm | 83 (34.7) | 40 (41.2) |  |
| Biparietal diameter^b^ <67 mm | 14 (5.9) | 6 (6.2) |  |
|  |  |  |  |
| **Cortical GM score; median (range; IQR)** | **1 (0-7; 0-1)** | **0 (0-3; 0-1)** | **.02** |
| **Cortical GM classification; *n* (%)** |  |  | **.04** |
| Normal (0) | 104 (43.5) | 60 (61.9) |  |
| Mild (1) | 80 (33.5) | 21 (21.6) |  |
| Moderate (2) | 38 (15.9) | 8 (8.2) |  |
| Severe (≥3) | 17 (7.1) | 8 (8.2) |  |

|  | | | |
| --- | --- | --- | --- |
|  | **Utrecht cohort**  **(*n*=239)** | **St. Louis cohort**  **(*n*=97)** | ***P* Value** |
| ***Signal abnormalities; n (%)*** |  |  | **1.00** |
| None | 237 (99.2) | 97 (100) |  |
| Focal unilateral | - | - |  |
| Focal bilateral | - | - |  |
| Extensive unilateral | 2 (.9) | - |  |
| Extensive bilateral | - | - |  |
| ***Gyral maturation; n (%)*** |  |  | **<.001** |
| Delay <2 weeks | 140 (58.6) | 96 (99.0) |  |
| Delay 2-<4 weeks | 94 (39.3) | 1 (1.0) |  |
| Delay ≥4 weeks | 5 (2.1) | - |  |
| ***Increased extracerebral space; n (%)*** |  |  | **.42** |
| Interhemisperic distance <4 mm | 164 (68.6) | 61 (62.9) |  |
| Interhemisperic distance 4-<5 mm | 51 (21.3) | 20 (20.6) |  |
| Interhemisperic distance 5-<6 mm | 13 (5.4) | 8 (8.3) |  |
| Interhemisperic distance ≥6 mm | 11 (4.6) | 8 (8.3) |  |
|  |  |  |  |
| **Deep GM score; median (range; IQR)** | **0 (0-3; 0-0)** | **0 (0-4; 0-1)** | **<.001** |
| **Deep GM classification; *n* (%)** |  |  | **<.001** |
| Normal (0) | 221 (92.5) | 52 (53.6) |  |
| Mild (1) | 14 (5.9) | 33 (34.0) |  |
| Moderate (2) | 1 (.4) | 7 (7.2) |  |
| Severe (≥3) | 3 (1.3) | 5 (5.2) |  |
| ***Signal abnormalities; n (%)*** |  |  |  |
| None | 223 (93.3) | 92 (94.9) | **.13** |
| Focal unilateral | 12 (5.0) | 2 (2.1) |  |
| Focal bilateral | 1 (.4) | 2 (2.1) |  |
| Extensive unilateral | 3 (1.3) | - |  |
| Extensive bilateral | - | 1 (1.0) |  |
| ***Volume reduction; n (%)*** |  |  | **<.001** |
| Deep GM area^b^ ≥9.5 cm^2^ | 237 (99.2) | 53 (54.6) |  |
| Deep GM area^b^ 8.5-<9.5 cm^2^ | 2 (.9) | 35 (36.1) |  |
| Deep GM area^b^ 7.5-<8.5 cm^2^ | - | 8 (8.3) |  |
| Deep GM area^b^ <7.5 cm^2^ | - | 1 (1.0) |  |
|  |  |  |  |
| **Cerebellum score; median (range; IQR)** | **0 (0-7; 0-1)** | **1 (0-7; 0-2)** | **<.001** |
| **Cerebellum classification; *n* (%)** |  |  | **<.001** |
| Normal (0) | 143 (59.8) | 42 (43.3) |  |
| Mild (1) | 58 (24.3) | 23 (23.7) |  |
| Moderate (2) | 24 (10.0) | 13 (13.4) |  |
| Severe (≥3) | 14 (5.9) | 19 (19.6) |  |
| ***Signal abnormalities; n (%)*** |  |  | **.20** |
| None | 198 (82.8) | 74 (76.3) |  |
| Punctate unilateral | 24 (10.0) | 10 (10.3) |  |
| Punctate bilateral | 10 (4.2) | 5 (5.2) |  |
| Extensive unilateral | 5 (2.1) | 4 (4.1) |  |
| Extensive bilateral | 2 (.8) | 4 (4.1) |  |
| ***Volume reduction; n (%)*** |  |  | **<.001** |
| Transcerebellar diameter^b^ ≥50 mm | 167 (69.9) | 43 (44.3) |  |
| Transcerebellar diameter^b^ 47-<50 mm | 50 (20.9) | 31 (32.0) |  |
| Transcerebellar diameter^b^ 44-<47mm | 16 (6.7) | 18 (18.6) |  |
| Transcerebellar diameter^b^ <44 mm | 6 (2.5) | 5 (5.2) |  |
| ^a^ partial corpus callosal thinning: genu <1.3mm and/or body <1.3mm and/or splenium <2.0mm; global corpus callosal thinning: genu <1.3mm and body <1.3mm and splenium <2.0mm;  ^b^ corrected for PMA. | | | |
