# Supplementary material for: Preterm brain injury on term-equivalent age MRI in relation to perinatal factors and neurodevelopmental outcome at two years
Source: PLoS One. 2017 May 9;12(5):e0177128. doi: 10.1371/journal.pone.0177128 (PMC5423624; doi:10.1371/journal.pone.0177128)
Supplement: S2 Table — (DOCX) [file pone.0177128.s003.docx]

| **S2 Table.** Distribution of brain metrics incorporated in the TEA-MRI scoring system across the Utrecht and St. Louis cohorts. | | | |
| --- | --- | --- | --- |
| **Brain metrics; median (range)** | **Utrecht cohort**  **(*n*=239)** | **St. Louis cohort**  **(*n*=97)** | ***P* Value** |
| Biparietal diameter^a^ (mm) | 72.7 (63.4; 86.2) | 72.2 (56.8; 82.2) | .40 |
| Corpus callosum: genu (mm) | 1.7 (1.1; 5.0) | 1.7 (1.0; 3.5) | .91 |
| Corpus callosum: body (mm) | 1.2 (.6; 2.4) | 1.4 (.9; 2.0) | <.001 |
| Corpus callosum: splenium (mm) | 2.4 (1.2; 4.2) | 2.2 (1.2; 4.4) | .13 |
| Right atrial width (mm) | 5.7 (2.3; 17.6) | 7.7 (2.9; 13.3) | <.001 |
| Left atrial width (mm) | 5.9 (3.0; 17.7) | 7.7 (2.5; 18.5) | <.001 |
| Interhemispheric distance (mm) | 3.2 (.0; 7.9) | 3.4 (.5; 8.8) | .02 |
| Deep GM area^a^ (cm^2^) | 11.5 (9.4; 13.7) | 9.6 (7.0; 12.8) | <.001 |
| Transcerebellar diameter^a^ (mm) | 51.5 (24.8; 58.1) | 49.6 (38.8; 55.1) | <.001 |
| ^a^ corrected for PMA. | | | |
